# Portable multi-focal visual evoked potential diagnostics for multiple sclerosis/optic neuritis patients

**DOI:** 10.1007/s10633-024-09980-z

**Published:** 2024-07-03

**Authors:** S. Mohammad Ali Banijamali, Craig Versek, Kristen Babinski, Sagar Kamarthi, Deborah Green-LaRoche, Srinivas Sridhar

**Affiliations:** 1 Department of Mechanical and Industrial Engineering, Northeastern University, Boston, MA USA; 2 NeuroFieldz Inc, Newton, MA USA; 3 Department of Neurology, Tufts Medical Center, Tufts University School of Medicine, Boston, MA USA; 4 Department of Physics, Department of Bioengineering and Department of Chemical Engineering, Northeastern University, Boston, MA 02115 USA

**Keywords:** Multi-focal visual evoked potential (mfVEP), Full-field visual evoked potential (ffVEP), Multiple sclerosis (MS), Optic neuritis, Machine learning, Signal processing, Portable diagnostics, Support vector machine (SVM)

## Abstract

**Purpose:**

Multiple sclerosis (MS) is a neuro-inflammatory disease affecting the central nervous system (CNS), where the immune system targets and damages the protective myelin sheath surrounding nerve fibers, inhibiting axonal signal transmission. Demyelinating optic neuritis (ON), a common MS symptom, involves optic nerve damage. We’ve developed NeuroVEP, a portable, wireless diagnostic system that delivers visual stimuli through a smartphone in a headset and measures evoked potentials at the visual cortex from the scalp using custom electroencephalography electrodes.

**Methods:**

Subject vision is evaluated using a short 2.5-min full-field visual evoked potentials (ffVEP) test, followed by a 12.5-min multifocal VEP (mfVEP) test. The ffVEP evaluates the integrity of the visual pathway by analyzing the P100 component from each eye, while the mfVEP evaluates 36 individual regions of the visual field for abnormalities. Extensive signal processing, feature extraction methods, and machine learning algorithms were explored for analyzing the mfVEPs. Key metrics from patients’ ffVEP results were statistically evaluated against data collected from a group of subjects with normal vision. Custom visual stimuli with simulated defects were used to validate the mfVEP results which yielded 91% accuracy of classification.

**Results:**

20 subjects, 10 controls and 10 with MS and/or ON were tested with the NeuroVEP device and a standard-of-care (SOC) VEP testing device which delivers only ffVEP stimuli. In 91% of the cases, the ffVEP results agreed between NeuroVEP and SOC device. Where available, the NeuroVEP mfVEP results were in good agreement with Humphrey Automated Perimetry visual field analysis. The lesion locations deduced from the mfVEP data were consistent with Magnetic Resonance Imaging and Optical Coherence Tomography findings.

**Conclusion:**

This pilot study indicates that NeuroVEP has the potential to be a reliable, portable, and objective diagnostic device for electrophysiology and visual field analysis for neuro-visual disorders.

## Introduction

Multiple sclerosis (MS) is a neuroinflammatory disease that damages the myelin sheath, nerve cell bodies, and axons. The resulting lesions may be found throughout the spinal cord and the brain. Optic neuritis (ON) is often a manifestation of MS and involves inflammation of the optic nerve, causing partial or complete loss of vision usually in one eye. About 1 in 5 MS patients experience ON as their initial symptom, and 50% of people with MS experience ON during the course of their disease [1]. More than 2.3 million people are thought to have MS worldwide, and the disease has impacted more than 400,000 people in the USA [2].

Visual evoked potentials (VEPs) are electrophysiological signals in response to visual stimuli that are retrieved from the electroencephalographic activity in the visual cortex and are recorded using scalp electrodes. Responses from a full-field VEP (ffVEP) test, which appear significantly delayed compared to normal, have long been recognized as a biomarker for diagnosing MS [3]. The peak time of the P100 component of ffVEPs provides reliable and objective information about the integrity of the visual system and proof of demyelination in MS patients but is limited in terms of providing spatially localized information about the visual field. The multi-focal visual evoked potentials (mfVEP) technique enables the simultaneous recording of VEPs from a large number of visual field regions in a time much shorter than sequential methods, allowing for the identification of spatially localized retinal damage as well as the lateralization of optic nerve and cortical dysfunction with the use of multi-channel recording montages [4]. This technique has proven applications in the assessment of visual field disorders and the diagnosis of diseases like optic neuritis/multiple sclerosis and glaucoma [5–7]. ffVEP quality can provide a useful guide to interpreting the more complex set of mfVEP signals; therefore, we hypothesized that combining these two methods would yield a more powerful diagnostic tool than either alone.

Utilizing a custom mobile virtual reality (VR) headset and in-house developed neuroelectric sensing hardware, we have developed a portable wireless system, called NeuroVEP, that enables the combined ffVEP and mfVEP tests. Using a mobile phone display, stimuli are presented to each eye separately while the other is held in darkness. An array of eight proprietary hydrogel-encapsulated electrodes senses the brain’s responses in the vicinity of O1, Oz, O2, O9, and O10 scalp locations (extended 10–20 system). Recording from multiple locations allows for the lateralization of ffVEPs coming from the left and right hemispheres and helps with improving the signal-to-noise ratio (SNR) for mfVEP results. Using the Scientific Python and Scikit Learn platform [8, 9], we developed an automated signal processing, statistical analysis, and machine-learning framework, which handles our recorded EEG signals and stimulus timing events for the combined ffVEP and mfVEP testing paradigms.

The device was tested on a group of subjects with MS and/or ON and a control group of normally sighted subjects. The ffVEP results were compared to a standard of care (SOC) device for ffVEP testing, Natus Nicolet, used by our clinical partners. Unlike the NeuroVEP which is portable, the Natus system is cart-based; furthermore, the SOC’s electrodes are slightly invasive, requiring needles to be inserted into the subject’s scalp. The use of needle electrodes is not common practice, however. Surface electrodes have been widely and successfully used in other experimental and clinical setups.

## Materials and methods

### Subject pool

This study was approved by the Northeastern University and Tufts Medical Center Institutional Review Boards (IRB Study # 13,395, Protocol title: Objective Portable Diagnostics of Neurological Disorders using Visual Evoked Potentials) and was performed in accordance with the Declaration of Helsinki. All subjects were either referred by their clinical neurologist at the Department of Neurology at Tufts Medical Center or were self-enrolled after seeing an advertisement posted around Tufts Medical Center. Subjects were required to be between 18 and 80 years of age and signed a written informed consent document after the study was explained and all their questions were answered. Gender was not used as a condition for selection. 10 healthy subjects along with 10 subjects with MS, ON or both conditions were tested.

Because of the virtual reality (VR) capabilities of our display system, we required subjects to fill out a simulator sickness questionnaire; however, the static nature of our stimuli was not expected to induce motion sickness symptoms, and this was borne out in the results: no motion-sickness related discomfort was reported and all subjects preferred the NeuroVEP device over the standard of care device at Tufts Medical Center.

Three of the authors served as control subjects (SMAB, CV, and SS), who have extensive experience participating in EEG and/or psychophysical vision testing paradigms.

The ffVEP and mfVEP experiments were tested in 10 (× 2 eyes) Normal subjects and 10 (× 2) subjects with MS, ON or both. A detailed overview of subject characteristics is reported in the “Results and Discussion” section (see Table 5).

### Hardware

We have developed a wireless headset based on mobile virtual reality technology, where a smartphone with an OLED screen displays stimuli while an array of eight neuroelectric sensors attached to the rear of the headset captures the subject’s responses from the visual cortex. We are using an updated prototype similar to the one that was developed in our lab for the diagnosis of age-related macular degeneration (AMD) [10, 11]; the modifications include: a more ergonomic fit, decreased weight, and better ventilation for the headset; the incorporation of a commercial eye-tracking device (Pupil Labs VR/AR add-on [12]); and a new generation of our custom hydrogel encapsulated electrodes. (Refer to our patent US 11,701,046 BS [13], example 5 for disclosure of a previous generation electrode technology. The new generation of electrodes have a similar formulation, but do not contain magnesium or lithium salts, rather potassium and choline chlorides are used.)

### Headmount

The headset is primarily made of parts that were 3D-printed using a Formlabs Form 3 stereolithographic system. The design mounts a smartphone to the front using a switchable adapter plate and positions sensors on the back with an adjustable tension mechanism to allow consistent electrode contacts at precise scalp areas. A combination of rigid and flexible materials has been used to ensure a comfortable and light-tight fit on the head. The reduction of interior temperature and the headset’s overall weight were two other crucial factors that received particular attention. The headset also features a phototransistor sensor for accurate timing of stimulus onset and reversal as well as eye-tracking cameras to ensure the subjects’ compliance with test protocols.

### Neuroelectric sensing

**Fig. 1:**
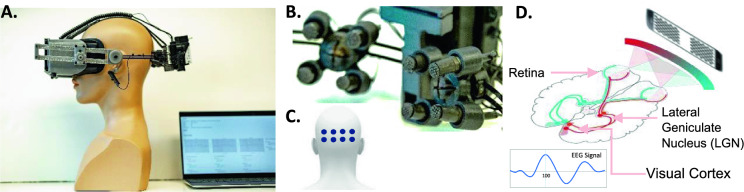
Portable wireless NeuroVEP system. **A** NeuroVEP device prototype integrating NeuroVEP sensor with visual stimulus headset, **B** Closeup of NeuroVEP EFEG sensor arrays, **C** Location of electrodes on the scalp over the visual cortex, **D** Dichoptic stimulus and typical neuroelectric response, Visual pathway. Image in part **D** Derived from (https://commons.wikimedia.org/wiki/File:Human_visual_pathway.svg)

The NeuroVEP system makes use of custom reusable hydrogel encapsulated electrodes that are both comfortable and convenient to set up. The tip of the soft yet mechanically robust hydrogel electrode (see Fig. 1B) is textured in order to hold a small layer of liquid electrolyte, which penetrates through scalp hair, providing a stable and low impedance contact. The tip diameter is between 6 and 7 mm, smaller than standard 10 mm EEG cup electrodes. About 10 min prior to setting the headset on the subject, the electrolyte is swabbed directly onto the electrode tips. Then, after adjusting the headset tension, impedance is measured for each channel and additional electrolyte is swabbed on the subject’s scalp around the contact sites, while hair is moved aside: we have previously established [14] that contact impedances (at 30 Hz) below 150 kOhms give adequate low noise levels, where values of 50–100 kOhm after touch-ups are typical. The scalp channels are referenced to the left ear lobe and the patient ground (bias) electrode is placed at the right ear lobe, both using custom hydrogel ear clip electrodes.

### Visual stimuli

There are two sections to the testing paradigm: a short 2.5-min ffVEP test is followed by a longer 12.5-min mfVEP test; they are separated by a 30-s break period, making the total length 15.5 min. For the ffVEP, 90 trials are recorded for each eye, with intervals of 0.5 s plus a random 0 to 0.1 s between stimulations; the set of trials are broken into 6 segments of 30 repetitions, switching eyes every new segment where there is a discarded onset/offset period of 2 s. The mfVEP test has a total of 16,384 overlapping trials at a rate of 60 frames per second; the sequence is divided into 16 segments of 1024 trials, switching eyes every new segment with a similar onset/offset period at the start.

The Google Pixel 2 XL smartphone’s OLED screen combined with optics that resemble the Google VR Daydream View (first version, 2016) was used for displaying the stimulus. Given the light-tightness of our headset and minimal reflected light in the viewer, the OLED screen on the phone offers an effectively infinite contrast ratio. The stimuli patterns were generated as high-resolution pixel maps (2048 × 2048) using custom Python programs, then were loaded into a custom app using the Google VR Android SDK with OpenGLES2 texture rendering.

**Fig. 2:**
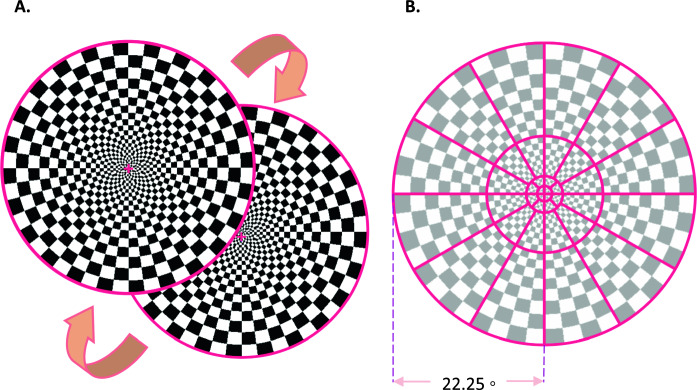
**A** ffVEP dartboard pattern-reversal visual Stimulus, **B** mfVEP 36 sectors visual stimulus

The mfVEP stimulus is similar to a dartboard that extends from 0° to 22.25° eccentricity and is broken into 36 visual field subregions [15] (Fig. 2B). The sector sizes are scaled based on cortical magnification factors to produce relatively similar areas of cortical stimulation according to the methods of Baseler et al. [4] which produces a 60-sector pattern with six rings of eccentricity; in order to derive the 36 sectors used herein we have merged rings 3 with 4 and 5 with 6. During stimulation, sectors are reversed (or not) simultaneously according to 36 pseudorandom uncorrelated binary sequences at 60 frames per second; individual responses are extracted using a correlation between each sector sequence’s time markers and the continuous EEG recording, known as the m-sequence technique [16]. For comparative purposes, the ffVEP stimulus uses the same geometry dartboard as mfVEP, but the whole pattern reverses at the same time (Fig. 2A)—we note that this arrangement is not standard clinical practice for transient pattern reversal VEP, where square checks of uniform size are more often used. Subjects were instructed to wear any spectacles or contact lenses if they are needed to have their best corrected visual acuity.

A hidden patch of the screen is toggled between black and white to trigger the phototransistor sensor to mark the stimulus reversal events on the same clock as the EEG samples; epochs are defined from the start of the trigger event up to 500 ms. As the Pixel 2 XL OLED display updates, changes in pixels sweep across the screen from the left side to the right (when in the standard VR mode horizontal orientation). The timing delays for most of the mfVEP sectors for both the left and right sides of the display were measured against the event signaling patch using a dual phototransistor setup. The average delay of the left center was found to be − 3.3 ms, and that of the right center was + 4.36 ms; overall, the delay was found to vary linearly as a function of horizontal pixel coordinate. These central values were used to correct the ffVEP events—otherwise, there would have been a significant error bias of around + 8 ms in peak time (latency) measures between right and left eyes. A linear model (R^2^ >  = 0.99) for each screen side was used to correct each mfVEP sector’s events using its average horizontal pixel coordinate.

### Data analysis

The EEG recording (at 1000 samples per second) and paradigm event data are streamed wirelessly to a computer as it is acquired and are cached into a file based on the Advanced Scientific Data Format (ASDF) [17], along with subject metadata and electrode impedance measurements. On the computer, an automated data analysis framework does the work of post-processing, analysis, and classification separately on the ffVEP and mfVEP signals.

Before subsequent data processing, each channel is prefiltered using a Stationary Wavelet Transform (SWT) baseline removal with an effective cutoff frequency of 0.5 Hz; this step reduces the effect of impulse response artifacts in subsequent filtering steps [10]. For this study, where the responses will be primarily analyzed using machine learning algorithms that may be sensitive to excess noise, we made the choice to reduce the frequency content to the band of 3–13 Hz (which includes P100 deflections as well as intrinsic alpha waves), using a Bessel high-pass IIR and an SWT low-pass filter. It is important to recognize that bandwidth reduction will inevitably create some distortion of time domain features, such as the peak time of the P100 components; however, many other experimental aspects, such as the details of the stimuli presentation will also affect the distributions of response parameters—this is precisely why each laboratory or device system must have its own set of normative data to compare against [18, 19].

### Full-field neuro-responses signal processing

**Fig. 3:**
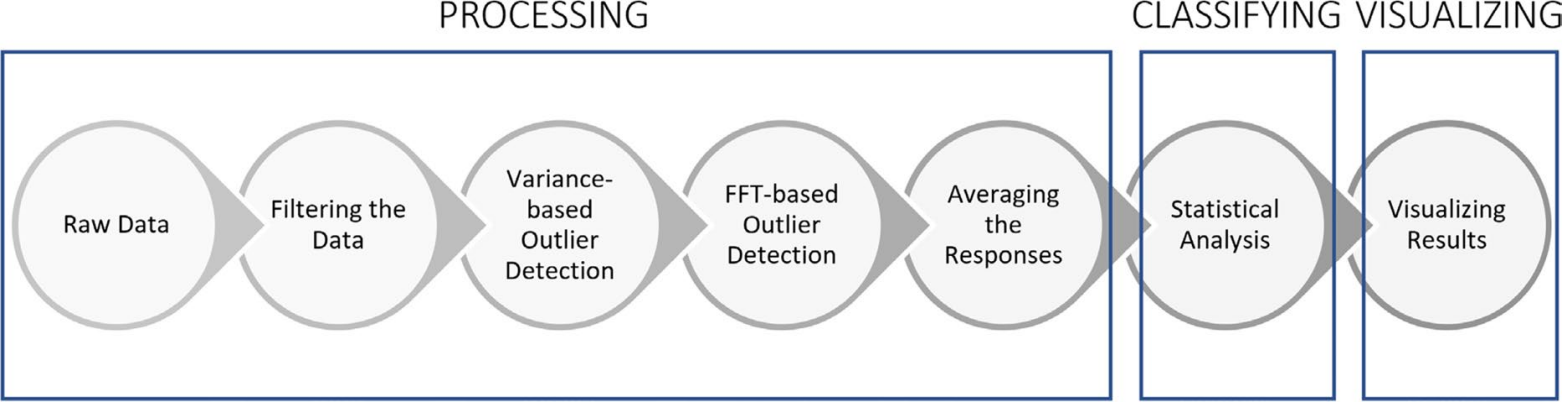
Full-Field stimulus data analysis steps

**Fig. 4:**
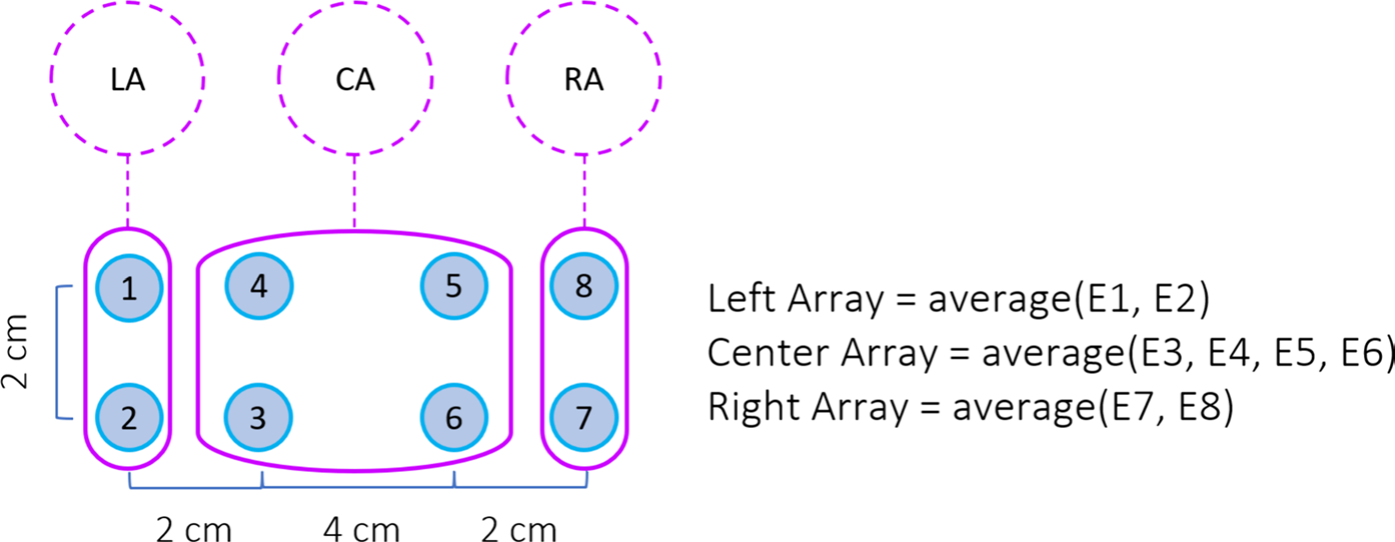
Arrangement of the electrodes and calculation of left, center, and right array responses

The steps involved in the processing of the full-field responses are shown in Fig. 3. The raw data initially undergoes the bandwidth reduction filters described above. Then, an unsupervised machine learning outlier rejection algorithm (Scikit-Learn’s “Isolation Forest” model [20]) excludes trials with outlier variances which are mainly caused by movement artifacts. Next, an alpha wave sensitive outlier rejection algorithm evaluates a fast Fourier transform (FFT) on individual trials and excludes those with power predominantly within the alpha frequency band (9–12 Hz)—rejections typically constitute a small fraction of the total trials. It has been shown that alpha waves are amplified during resting, sleeping, eye closures and, in our experience, when the subject is unable to see the stimulus; conversely, alpha waves are attenuated by visual attention [21–23]. Then, the selected trials recorded from each electrode are averaged across time to form 8 individual response channels. Using these 8 signals we then calculate responses from the three scalp locations: the left hemisphere is the average of electrodes 1 and 2 (O1), the right hemisphere is the average of electrodes 7 and 8 (O2) and the central signal is calculated by averaging electrodes 3, 4, 5 and 6 (Oz) (Fig. 4).

The first major positive component of the full-field VEP, P100, has been shown to be delayed or absent in patients with MS or ON [3, 24, 25]. We measure the peak time and amplitude of P100 components of individual eyes at three different scalp locations (O1, Oz, and O2). We also calculate the interocular and interhemispheric peak time difference and amplitude ratios. Waveform and amplitude abnormalities also have been considered biomarkers for MS and ON [26].

### Full-field classification and visualization

**Fig. 5:**
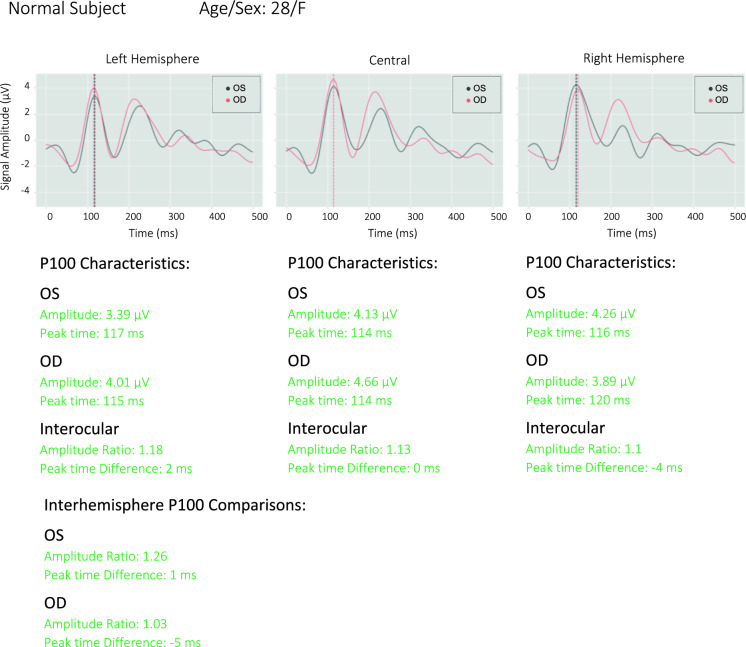
Full-field test reports of a representative normal subject from NeuroVEP Device

Figure 5 shows an example of a full field report. The data are presented separately for each eye and for three separate scalp locations. Data from the left hemisphere is the average of electrodes 1 and 2 (O1), the central location is the average of electrodes 3, 4, 5, and 6 (Oz), and the data from the right hemisphere is the average of electrodes 7 and 8.

6 distinct parameters have been investigated for the ffVEP signals: peak time (latency), amplitude, interocular peak time difference, interocular amplitude ratio, interhemispheric peak time difference, and interhemispheric amplitude ratio. Each of these parameters may indicate dysfunctions in the prechiasmatic, chiasmatic, or postchiasmatic regions of the visual field pathway.

Latency-based biomarkers are less sensitive to retinal and ocular diseases and more reliable in examining visual pathways compared to amplitude-based ones. However, in the absence of other retinal diseases, abnormal peak time delays generally signify demyelination whereas abnormally reduced amplitudes are a sign of axonal loss [19].

A monocular delayed peak time suggests a dysfunction in the optic nerve on one side. Conversely, if there is a bilateral abnormality in peak time, it indicates a dysfunction in the visual pathway on both sides, but determining whether it is located in pre or post-chiasmatic regions would require additional evaluation of amplitude and topographic (visual field mapping) features [27].

Amplitude-based metrics of P100 are much more sensitive to ocular and retinal disease. Patient factors such as poor fixation, loss of focus, tearing, inattention, or drowsiness can all cause a decrease in mid-occipital amplitude. However, after ruling out these factors, a monocular amplitude abnormality suggests a dysfunction in the visual pathway of one eye before the optic chiasm. A bilateral abnormality indicates bilateral disease, but a more detailed analysis of topographic features or responses to partial field stimulation is necessary to localize the specific site affected beyond the post-chiasmal region. Testing one or both eyes may reveal lateral occipital amplitude asymmetries (interhemispheric amplitude ratio abnormalities). In the absence of P100 peak time or interocular amplitude abnormalities, such asymmetries are not necessarily of clinical importance. However, in the presence of the mentioned abnormalities, they may be the sole indication of dysfunction in the chiasmal or post-chiasmal visual pathway. Typically, such abnormalities are present in both eyes. If the asymmetry is unilateral, it may suggest a partial dysfunction before the optic chiasm.

To evaluate all parameters, each laboratory or device must establish its own unique normative values [28]. Independently to data collected in this study, we tested the same implementation of ffVEP on a group of 13 normally sighted subjects (26 eyes) with a mean age of 33 ± 7 years to establish normative classification criteria for all parameters; we note that three of the same subjects, namely the authors (s1, s2, s3), appear in both the normative database and controls group of this study, but separate recordings were used. Based on the following criteria, each parameter is classified as normal (green), borderline (yellow), or abnormal (red), where *std* refers to its standard deviation: for all latency-based metrics, including interocular and interhemispheric peak time differences, a value below “mean + 2 *std”* was marked as normal, between 2 and 3 *std* from the mean was marked as borderline, and above 3 *std* away from the mean was marked as abnormal. For all amplitude values, a natural logarithm of one plus the distribution (*ln(amp*_*i*_ + *1)*) was first calculated to get a more normal distribution, then similarly, any nonexistent (or negative amplitude) P100 or values below “mean—3 *std”* were marked as abnormal, between “mean—3 *std”* and “mean—2 *std”* were marked as borderline and above “mean—2*std”* were marked as normal. This procedure has been recommended by the American Clinical Neurophysiology Society Guidelines on Visual Evoked Potentials [27]. The main ffVEP statistics for the normative subject pool are summarized in Table 1.

**Table 1:** Normative statistics (from 13 subjects) of main ffVEP parameters on 3 scalp locations

Stat	LA PT	LA PT Diff	LA log(Amp)	CA PT	CA PT Diff	CA log(Amp)	RA PT	RA PT Diff	RA log(Amp)
Mean	110	5.08	1.36	105	3.31	1.61	107	4.77	1.36
Std	10.34	5.44	0.44	7.43	2.72	0.42	10.91	3.94	0.44

The logs of amplitudes are calculated as the natural logarithm of (1 + variable)

*LA* Left Array, *PT* Peak Time, *Diff* Difference, *CA* Central Array, *Amp* Amplitude, *RA* Right Array

All amplitude ratios were calculated as the larger value to the smaller value, then were classified based on the following thresholds: above 2.5 were marked as “abnormal”, between 2 and 2.5 were “borderline”, and less than 2 were “normal”.

### Signal processing of multi-focal neuro-visual responses

**Fig. 6:**
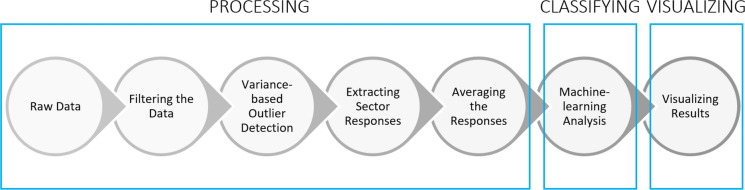
Multi-focal stimulus data analysis steps

Multifocal data analysis is more complicated. The steps involved in the data processing of mfVEP data are shown in Fig. 6. Similarly to ffVEP, the mfVEP data is filtered for bandwidth reduction (3–13 Hz) and the same outlier rejection algorithm marks trials for exclusion from response averages. Next, individual sector responses are extracted using the m-sequence technique [29].

**Fig. 7:**
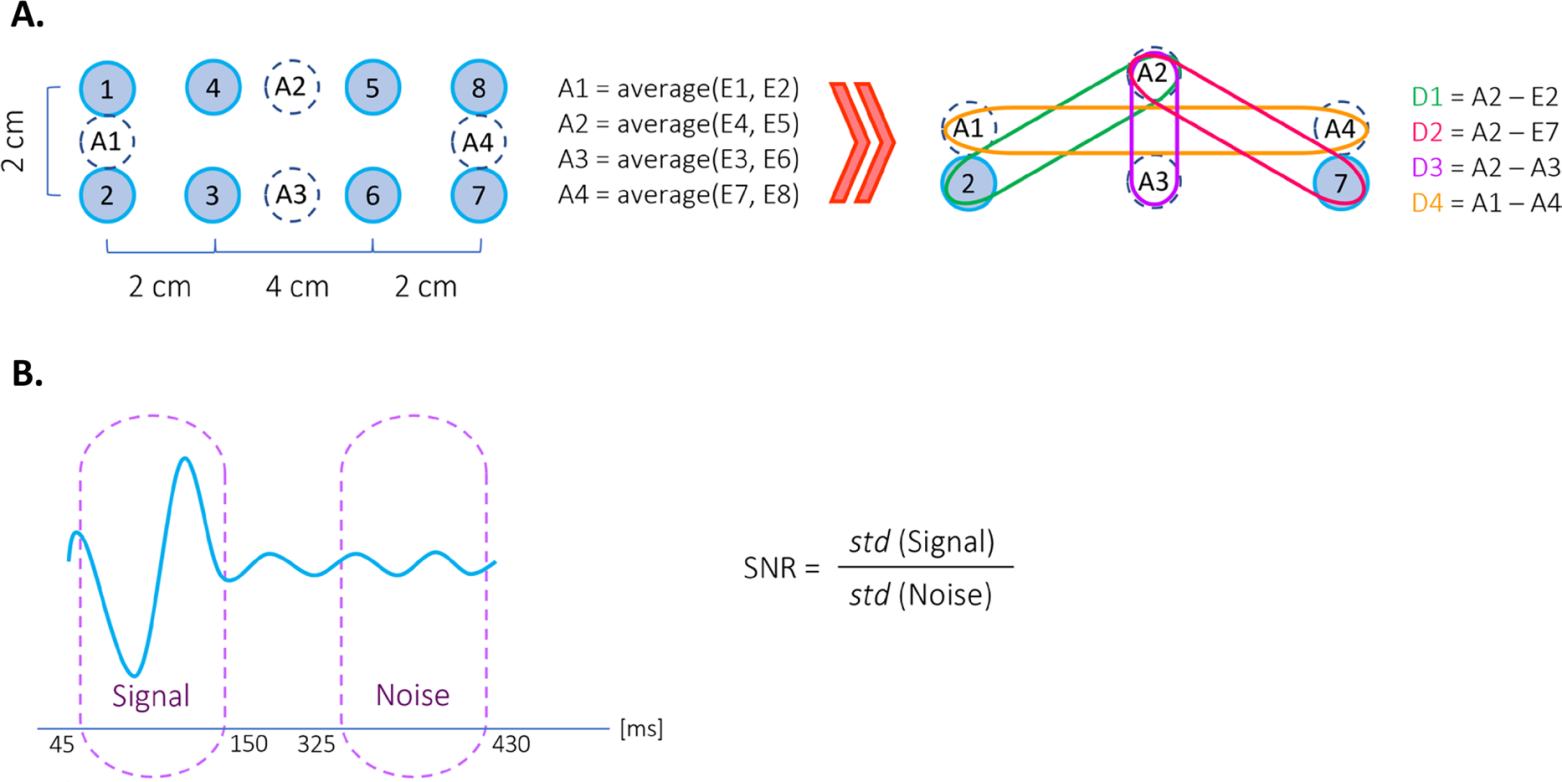
**A** Multi-focal test electrode arrangement and calculation of derived signals. **B** Calculation of SNR for mfVEP responses: Signal window [45:150] ms and Noise window [325:430] ms

We take advantage of recording from 8 individual sensors to increase the signal quality. The signals used for mfVEP are derived from the globally referenced (to the left ear clip electrode) channels, by taking pairwise subtractions of channel combinations that are closer together than they are to the reference; therefore, much of the signal in common which is due to non-local and unrelated brain processes or other electrophysiological artifacts gets canceled out. Additionally, given the variations in the folding of the brain’s visual cortex among individuals, it has been shown that recording from multiple locations and then combining the responses can significantly improve the SNR [30–32]. Figure 7A shows how derived signals D1, D2, D3, and D4 are computed from the original 8 channels. Based on our experience and recommendations from previous studies, these four combinations provided the highest SNR [30, 33]. Figure 7B shows the method of calculating the SNR values. Our selection of response window and noise window is similar to what was done in the previous studies [34]; however, our calculation of the SNR and noise window criteria is significantly different. Instead of using the average of noise windows of all sectors for each subject as the noise, we use the noise region of each individual sector of each subject for the calculation of the SNR. The earlier approach tends to yield higher SNR for the majority of responses due to the averaging of noise regions across a large number of responses, effectively minimizing variations in the noise window; consequently, this method diminishes the discriminative power of this parameter for the classification algorithm. The method we employed, on the other hand, more effectively distinguishes between the two classes, normal and abnormal (see next section). Finally, we compute the optimized response for each sector in the following manner: the derived signal responses are polarity matched to D2 and then are averaged together using their SNR as a weighting factor.

#### Multi-focal data classification

We have developed a machine learning framework for classifying and quantifying the responses. We have also created unique color maps to visualize mfVEP results.

#### Machine learning data set curation

For training the supervised machine learning algorithm (MLA), we started by manually selecting and labeling the most apparent sector responses from 7 different experiments (14 eyes) as “normal” and “abnormal”—a binary classification task. The selected experiments included 6 cases of healthy subjects with artificial defects introduced to the stimulus plus 1 case of an MS subject. The artificial defects were designed by partially or fully masking individual mfVEP sectors with black patches directly using the paradigm presentation software; we hypothesize that this masking would adequately simulate the effects of complete loss of vision in those regions. Responses from the masked regions or abnormal responses from the MS subject were labeled as “abnormal” and the healthy responses were labeled as the “normal” category. This initial dataset included 382 samples; however, we established that we could increase the accuracy of the machine learning algorithm by using data augmentation techniques to greatly increase the number of training samples. The training data was augmented to 65,536 (2^16^) samples using a randomized nearest-neighbor mixing and rescaling approach, where a random signal is selected and linearly interpolated with its nearest neighbor using a Gaussian random variable mixing factor centered around 0.5 with a standard deviation of 0.25, clipped to the range 0–1. We employed the KDtree algorithm [35] from the SciPy’s package for Python [9] for efficient nearest-neighbor identification.

**Fig. 8:**
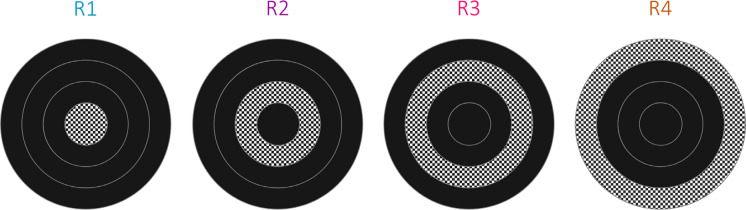
Schematic of the stimuli used for creating the “artificial defect” evaluation dataset. In each case, only sectors on one ring were unmasked (stimulating)

Despite sophisticated signal processing methods that perform well in filtering artifacts and alpha waves in some individuals, it has been observed that algorithms fail to avoid false positive or false negative classifications. This issue is not unique to mfVEP—the Humphrey Visual Field test can suffer a similar problem from subject participation errors. Other researchers have tried various methods to deal with these issues for monocular mfVEP test evaluation [36, 37]. Because of the risk of over-fitting, where MLA accuracy during training is much higher than when applied to new tests, the final evaluation of the system must not use data encountered during training. To evaluate the performance of both the MLA and the mfVEP test conducted by the NeuroVEP device, we built a separate dataset based on custom mfVEP stimuli with artificial defects (shown in Fig. 8) containing 576 samples from both eyes of 2 healthy volunteers (4 tests each, 16 tests total). In this case, the samples were not selected manually; instead, they were labeled based on whether the sector was masked or not.

#### Feature extraction

**Fig. 9:**
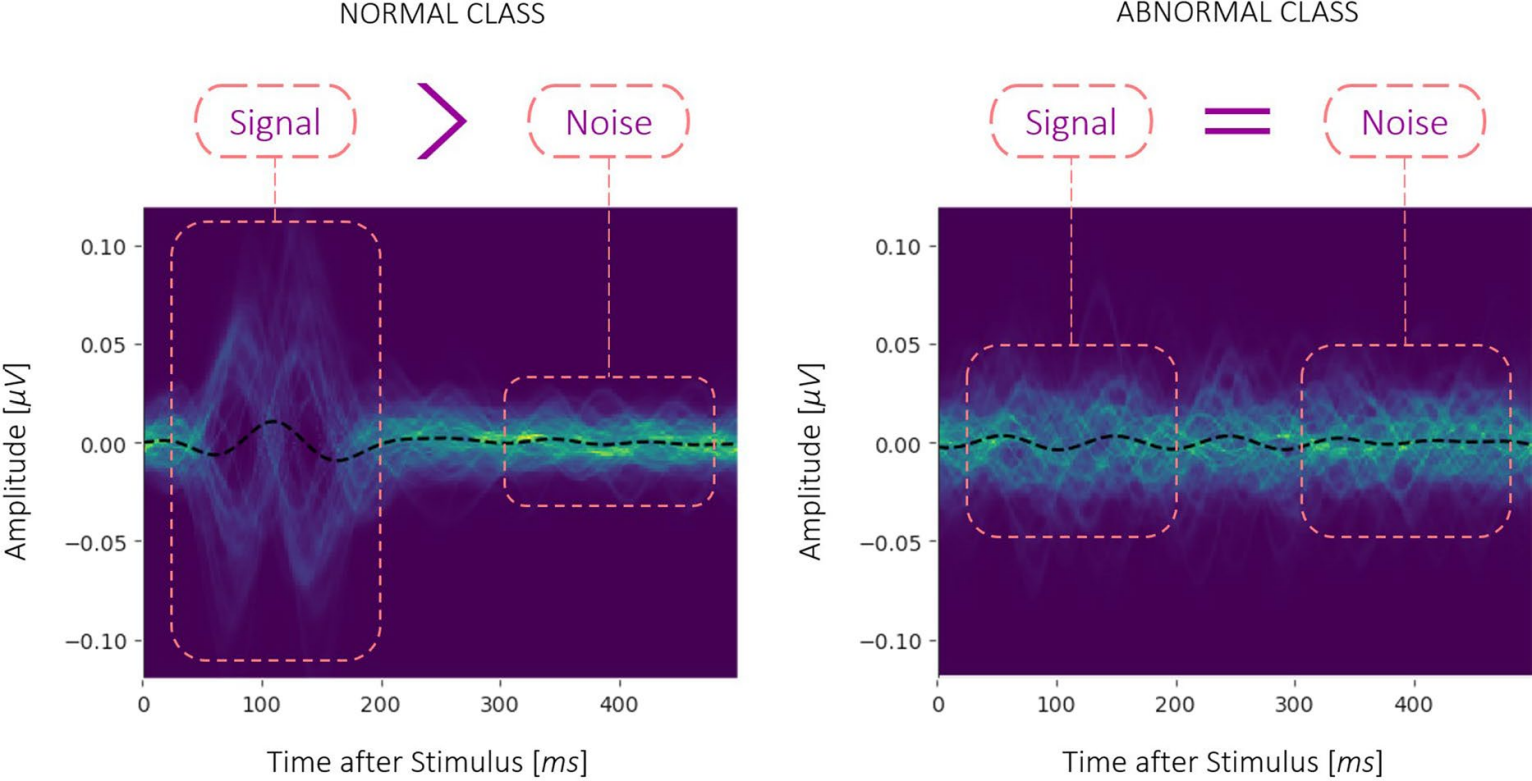
Overlay of training data **A.** Normal class versus **B.** Abnormal class

Feature extraction is a crucial step in developing a high-performance machine learning model. We started by extracting a large database of custom features which could be generally categorized into two types, Waveform-based and Frequency-based features. In the visual field regions where the subjects are unable to see (or just barely perceive) the stimulus, they usually produce responses within the frequency range of alpha waves (8–13 Hz); this effect can be seen in the —for "abnormal” responses (which are often indistinguishable from noise), the signal and noise windows that are used for SNR calculation look very similar to each other and contain higher amplitude alpha band content, than the same windows of “normal” responses. Figure 9 shows 2D histograms of the two classes of responses, where the overlaid curves are binned to produce a color-scaled “counts” dimension. The average over all example responses is shown with a black dashed curve.

Based on these considerations we kept 12 custom features that could effectively discriminate between the two classes of our machine learning problem. Later, we found that we could further improve the accuracy and performance of our model by introducing 7 more features from the well-known Catch-22 feature set [38]. These features belong to the following categories: Linear autocorrelation (CO_f1ecac and CO_FirstMin_ac), successive differences (MD_hrv_classic_pnn40, SB_BinaryStats_diff_longstretch0, and SB_MotifThree_quantile_hh) and simple temporal statistics (DN_OutlierInclude_p_001_mdrmd and DN_OutlierInclude_n_001_mdrmd). Therefore, for our final machine learning model, we used 19 features, listed in Table 2.

**Table 2:** List of features extracted from the signals for the machine learning training

Feature name	Definition
Custom Features	
sig_snr	Ratio of the std of Signal(LP 20 Hz)[50, 200] to Signal(LP 20 Hz)[250, 500] *ms*
sig-hp_cor	Correlation between Signal(BP)[0, 500] and Signal(HP 9 Hz)[0, 500] *ms*. It shows how much the signal is correlated with noise
response_cor	Correlation between Reference Normal Signal[0:200] and Other Signals(LP 20 Hz)[0:200] *ms*
std_ratio	Ratio of the std of the Signal(BP)[0:250] to Signal(HP 20 Hz)[250, 500] *ms*
mid_abs_auc_ratio	Ratio of AUC of ABS < Signal(LP 20 Hz)[0, 250] > /ABS < Signal(LP 20 Hz)[250, 500] > *ms*
skewness	Skewness of Signal(LP 9 Hz)[0, 500] *ms*
peak_amp_ratio_13	The ratio of the amplitude of the highest peak (positive or negative) to 3rd highest peak (positive or negative) on Signal(BP)[0, 500] *ms*
peak_amp_ratio_14	The ratio of the amplitude of the highest peak (positive or negative) to the 4th highest peak (positive or negative) on Signal(BP)[0, 500] *ms*
no_zero_x	The number of zero-crossings of Signal(BP)[0, 500] *ms*
fft_resp_to_alpha_power	The ratio of the power of the alpha frequency band of the signal [8–13 Hz] peak to the [0–8 Hz] frequency band peak, based on FFT analysis on the Signal(BP)[0, 500] *ms*
wv_apprx_std_0	std(first half of level 1 DWT approx. coefficients of the Signal(BP)[0, 500] *ms*.)
wv_apprx_std_1	std(first half of level 2 DWT approx. coefficients of the Signal(BP)[0, 500] *ms*.)
Catch-22 Features	
CO_f1ecac	On Signal(BP)[0:500] *ms*. First 1/e crossing of the autocorrelation function
CO_FirstMin_ac	On Signal(BP)[0:500] *ms*. First minimum of the autocorrelation function
MD_hrv_classic_pnn40	On Signal(BP)[0:500] *ms*. The proportion of successive differences exceeding 0.04σ
DN_OutlierInclude_p_001_mdrmd	On Signal(BP)[0:500] *ms*. Time intervals between successive extreme events above the mean
DN_OutlierInclude_n_001_mdrmd	On Signal(BP)[0:500] *ms*. Time intervals between successive extreme events below the mean
SB_BinaryStats_diff_longstretch0	On Signal(BP)[0:500] *ms*. The longest period of successive incremental decreases
SB_MotifThree_quantile_hh	On Signal(BP)[50:200] *ms*. Shannon entropy of two successive letters in equiprobable 3-letter symbolization

For the details of the Catch-22 features, please refer to its paper

*LP* low pass filter, *BP* Band pass [3–50 Hz] filter, *HP* high pass filter, *AUC* area under the curve, *ABS* absolute VALUE, *FFT* fast Fourier transfer, *DWT* discrete wavelet transform

#### Classification method

For the classification task, we compared several supervised ML algorithms: support vector machine (SVM), Logistic Regression, K-Nearest Neighbors (KNN), Naïve Bayes, Random Forest, Decision Tree, and ensemble meta-estimators AdaBoost and Bootstrap Aggregating (Bagging)—both of which used Decision Tree as the base estimator. Additionally, we tried a deep learning neural networks (NN) algorithm using just the response curves (rather than the hand-selected features) that used an architecture consisting of several stages of one-dimensional convolutional layers terminating with a densely connected classifier network—the highest accuracy that was attained by the NN approach was around 88%. In the end, we selected the SVM binary classifier as it produced the highest accuracy level and a balanced sensitivity versus specificity. SVM uses hyperplanes to separate the classes with maximal margins. Our model uses the Radial Basis Function (RBF) kernel to conform to the nonlinearities of the classification task.

Most models performed very well on the augmented training data set. For example, a fivefold cross-validation for the SVM model returns accuracies of [0.9983, 0.9979, 0.9978, 0.9981, 0.9975]. However, we selected our model based on its accuracy on the evaluation dataset. As discussed before, the accuracy of the evaluation set represents not only the accuracy of the model but also the NeuroVEP device, as it takes into account the inherent testing abnormalities such as subject fixation errors and residual movement artifacts. Table 3 shows the accuracy, sensitivity, specificity, and Area Under the receiver operating characteristic (ROC) Curve (AUC) for the models built using the training set and tested with the evaluation set.

**Table 3:** Performance comparison of different ML models on the evaluation dataset

Model	Accuracy	Sensitivity	Specificity	AUC
SVM	0.91	0.82	0.94	0.88
Logistic Regression	0.86	0.83	0.88	0.85
KNN	0.83	0.86	0.82	0.84
AdaBoost (Decision Tree)	0.79	0.9	0.76	0.83
Naive Bayes	0.77	0.9	0.73	0.81
Bagging (Decision Tree)	0.77	0.89	0.72	0.81
Random Forest	0.76	0.92	0.71	0.82
Decision Tree	0.71	0.92	0.65	0.78

Table 4 shows the effect of data augmentation and the addition of Catch-22 features on the test performance. For all these iterations, the SVM model with RBF kernel is used. It can be seen how making the model more complex step by step increased the accuracy from 0.8 to 0.91.

**Table 4:** The effect of data augmentation and Catch-22 features on the performance of the ML model. The symbol ‘✓’ denotes inclusion

Model	Data augmentation	12 Custom features	7 Catch-22 features	Accuracy	Sensitivity	Specificity	AUC
SVM	-	✓	-	0.8	0.91	0.76	0.84
SVM	-	✓	✓	0.83	0.85	0.83	0.84
SVM	✓	✓	-	0.84	0.9	0.82	0.86
SVM	✓	✓	✓	0.91	0.82	0.94	0.88

## Results

### Full-field VEP test

Table 5 summarizes all calculated metrics for the study’s subject pool. As discussed, the peak time of the P100 is the most robust biomarker for detecting visual pathway damage. Our analysis primarily looks at the peak time and peak time differences of the Oz location for each eye, as well as amplitude abnormalities of the waveform. If these parameters are in the normative range, the subject is labeled as “normal”. If the waveform and peak times of the Oz location were abnormal, we then used the information from the rest of the sensors at O1 and O2 locations to get a better understanding of the deficit. In some instances the waveforms were abnormal, so the interhemispheric (IH) or interocular (IO) ratios were marked as NA; for interhemispheric comparisons, the troubling results were reported as being in the left hemisphere (L), right hemisphere (R), or on both sides (B).

**Table 5:**
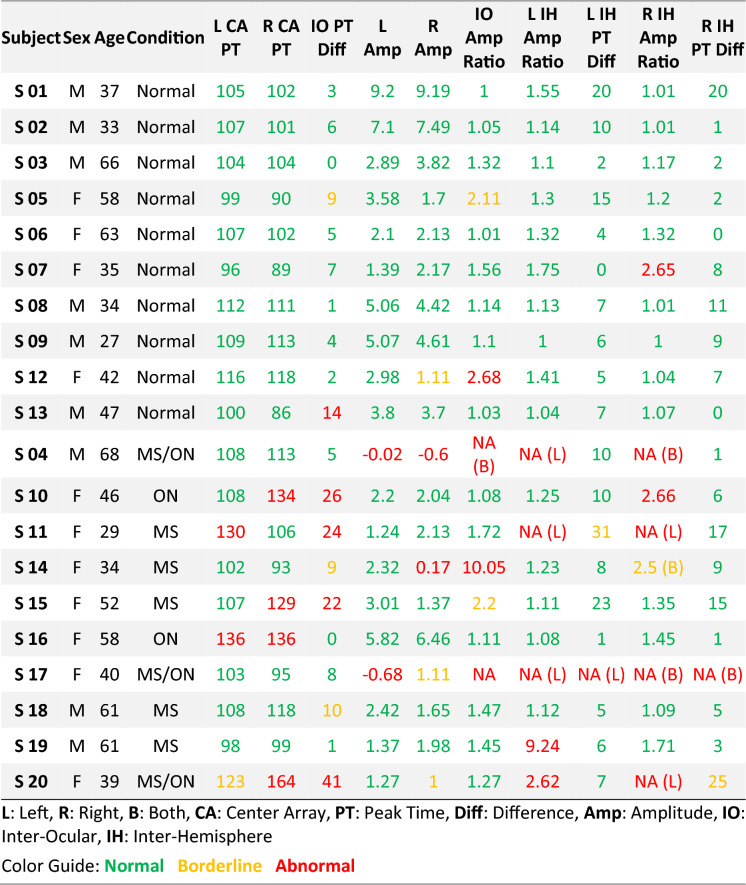
Summary of the results for all subjects

We compared our device’s ffVEP peak time measurements and diagnosis to those of the SOC VEP test being used in practice by our clinical partners at Tufts Medical Center, Boston, Massachusetts. The SOC tests were conducted by a skilled technician utilizing a Natus system (software version Nicolet EDX21.1; Natus Neurology, Pleasanton, CA) adhering to the guidelines set forth by the International Society of Clinical Electrophysiology of Vision (ISCEV) [19]. Participants were positioned 1 m from the computer screen and instructed to maintain visual fixation on a red cross positioned at the center of an alternating checkerboard pattern consisting of black and white squares (with a check size of 32 arcminutes) at 100% contrast. The recording was performed using a needle electrode at the Oz location; the technician determines the number of trials, adding more for weaker signals, and then hand-picks the P100 peaks for which the amplitude and peak time are reported—the waveforms are also included in the report. Finally, mainly based on the P100 peak times and to some extent the waveforms, a neurologist provides interpretation of the results. In Table 6, NeuroVEP’s diagnosis for all subjects is summarized along with the diagnosis using the SOC device.

**Table 6:**
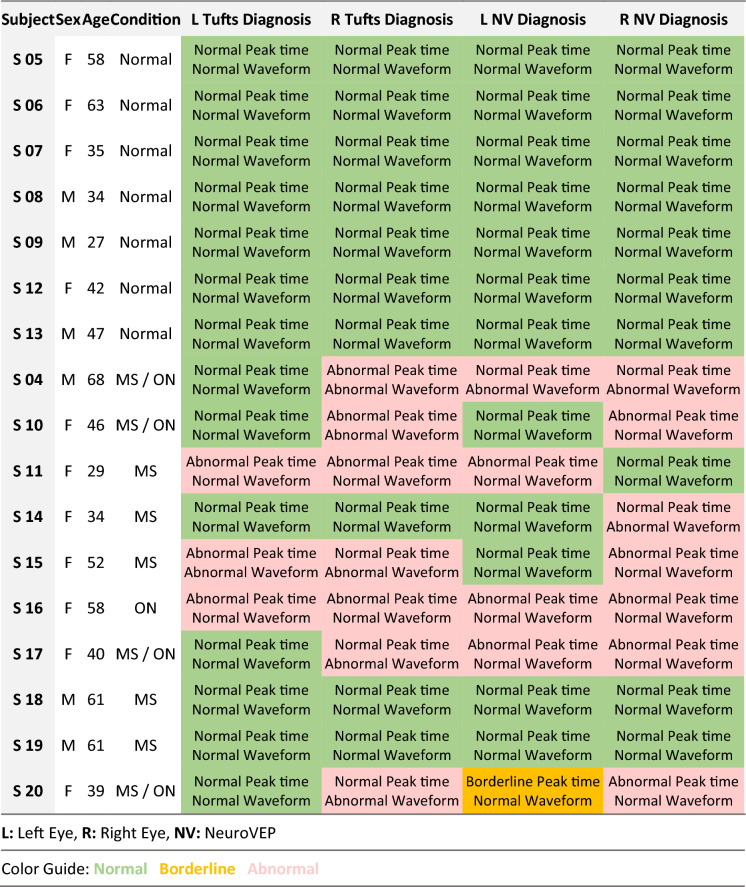
NeuroVEP versus tufts diagnosis results

**Fig. 10:**
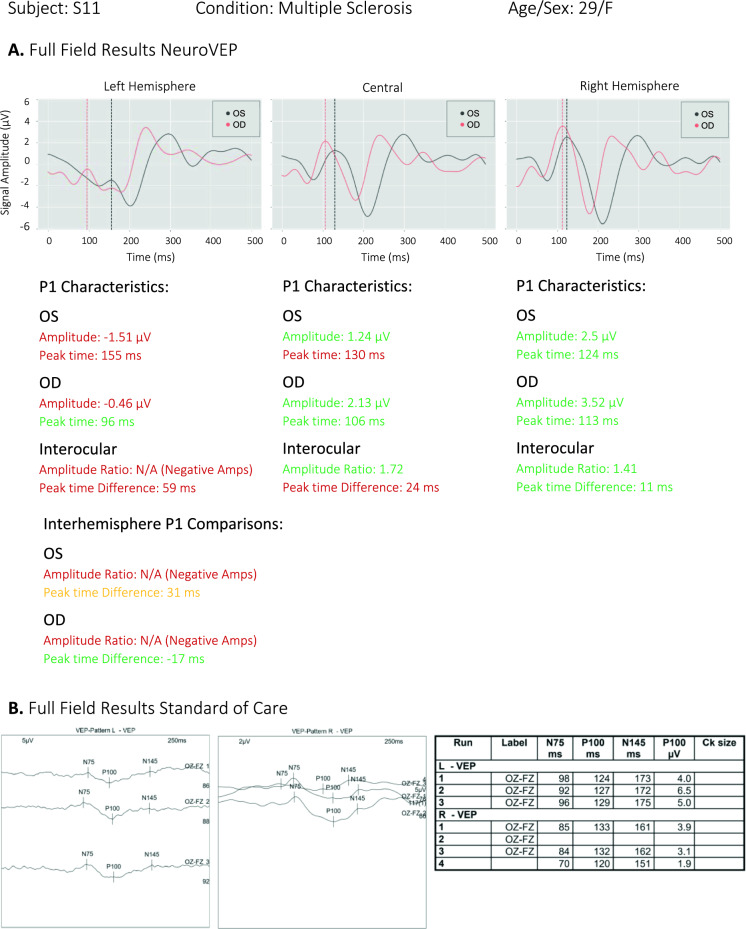
Full-field test reports of a MS/ON subject from** A** NeuroVEP Device (note, positive peaks point upward) and **B** Tufts SOC device (note, positive peaks point downward—as data was stored only as images, the presentation of these figures could not be improved)

In 31 of 34 eyes (91%), similar predictions were given by NeuroVEP and Tufts Diagnosis. However, the NeuroVEP device provides additional information afforded by recording from eight individual sensors. Also, based on the recorded subject’s feedback during the test, we believe NeuroVEP device provides more accurate and reliable measurements. For example, see Fig. 10A (S11/MS), where we can see both responses from the left and right eye on the left hemisphere (O1 Location) have abnormal waveforms (and therefore abnormal interhemispheric amplitude ratios). Because of the additional information here, the issue can be better explained with more detail as a lesion in the chiasmal or post-chiasmal regions of the visual field pathway. In Fig. 10B, the results of the same subject from the SOC device are shown. Here, the technician ran the test 3 times for the left eye; all three tests turned out to be successful. For the right eye, the technician ran the test 4 times, but only 3 tests were successful. The peak times are generally variable, and these results are then presented to a neurologist for comments.

### Multi-focal VEP test

**Fig. 11:**
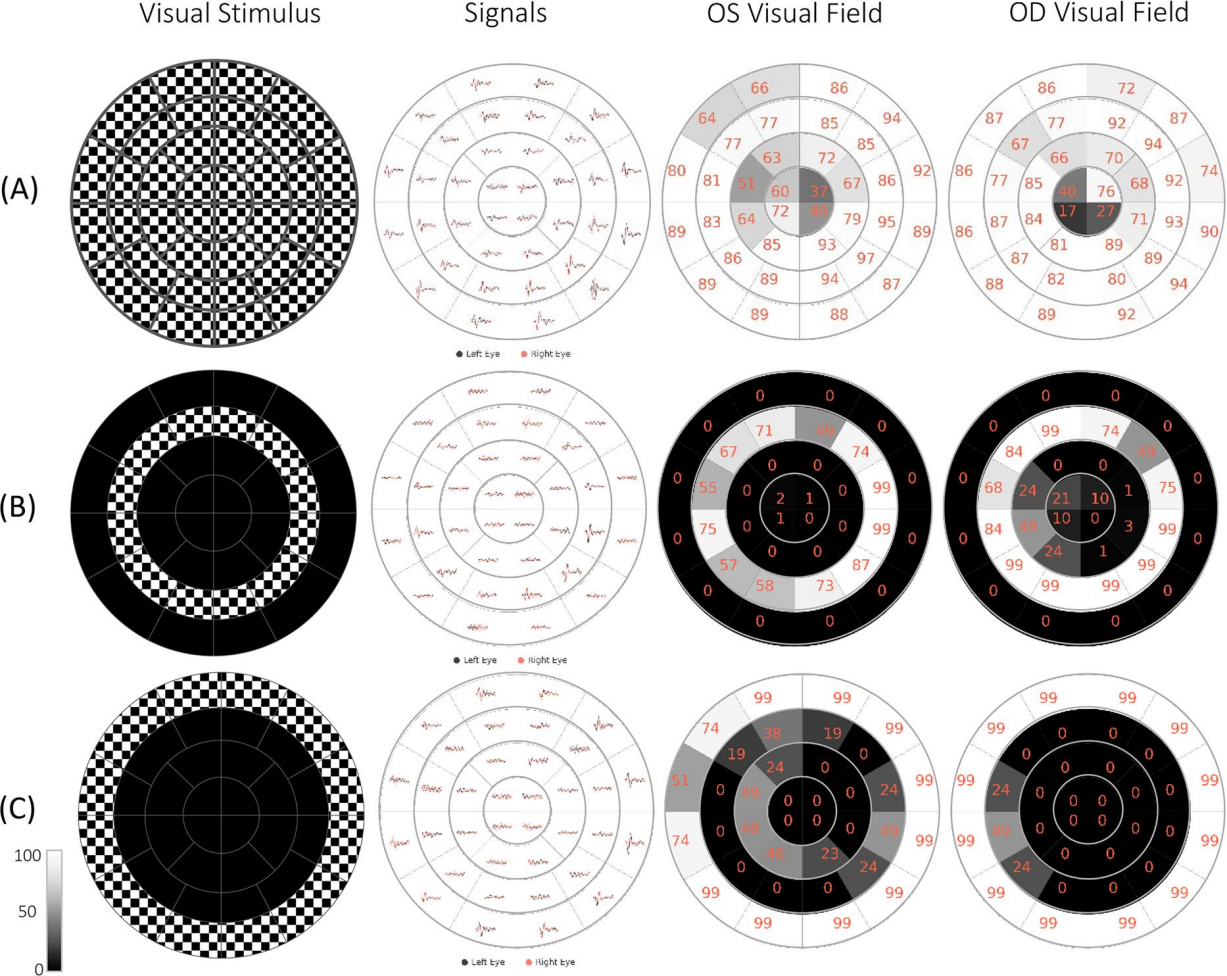
mfVEP custom stimuli, responses, and per eye visual function classifications for **A** full stimulus 0–22.25° ecc.** B** Artificially masked mid-peripheral ring stimulus 2.72–8.58° ecc., and **C** Artificially masked outermost peripheral ring stimulus 8.58–22.25° ecc. Scores between 0 and 100 represent the quality of the response based on the classification probabilities and are linearly gray-scaled (White: Normal, Black: Abnormal); therefore, mid-gray sectors may be marked as “ambiguous”, requiring further analysis

Figure 11 shows a sample of results for an unaltered stimulus and two different stimuli where defects were simulated (OS: Left eye and OD: Right eye). For each sector, a score between 0 and 100 is calculated based on the machine learning classification probabilities. Scores below 50 are deemed abnormal responses, and above 50 are deemed normal; these scores are used to quantify the sector responses using a grayscale map: whiter sectors are more certainly good responses, darker are more certainly poor responses (or the stimuli were artificially blocked), and mid-gray responses are ambiguous (should be reassessed by a trained technician).

**Fig. 12:**
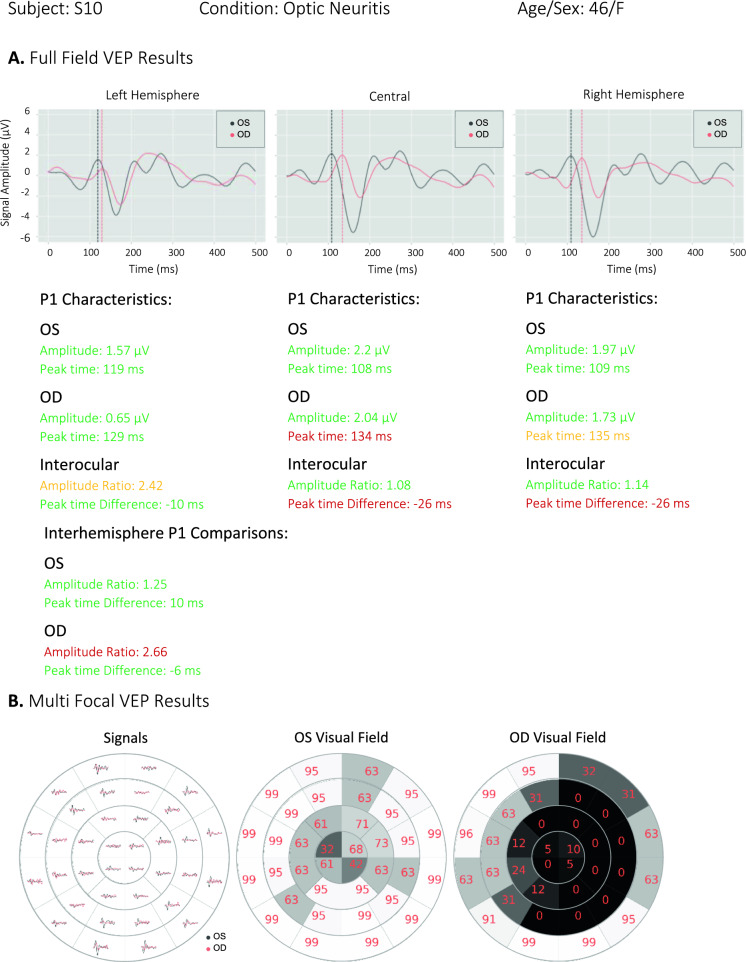
NeuroVEP’s results for subject S10 with right eye (OD) optic neuritis. **A** ffVEP results with delayed right eye (OD) and abnormal interhemispheric amplitude ratio,** B** mfVEP results which show a scotoma in the right eye

Figure 12 shows the ffVEP and mfVEP results of subject S10 with ON. The NeuroVEP’s ffVEP results show abnormal peak time for the right eye and an abnormal interhemispheric amplitude ratio. This suggests a dysfunction of the optic nerve on the right side which is probably prechiasmatic. The mfVEP test reveals either very poor vision or complete loss of vision in the dark areas of the right eye’s visual field. The pattern of visual field loss suggests a pre-chiasmatic dysfunction [39]. The NeuroVEP’s diagnosis matches with other clinical tests for this subject. The MRI shows an increased short tau inversion recovery (STIR) signal abnormality within the right optic nerve and associated enhancements. Optical coherence tomography (OCT) test for this subject reveals normal left eye but moderately severe thinning (except nasally) in the right eye.

**Fig. 13:**
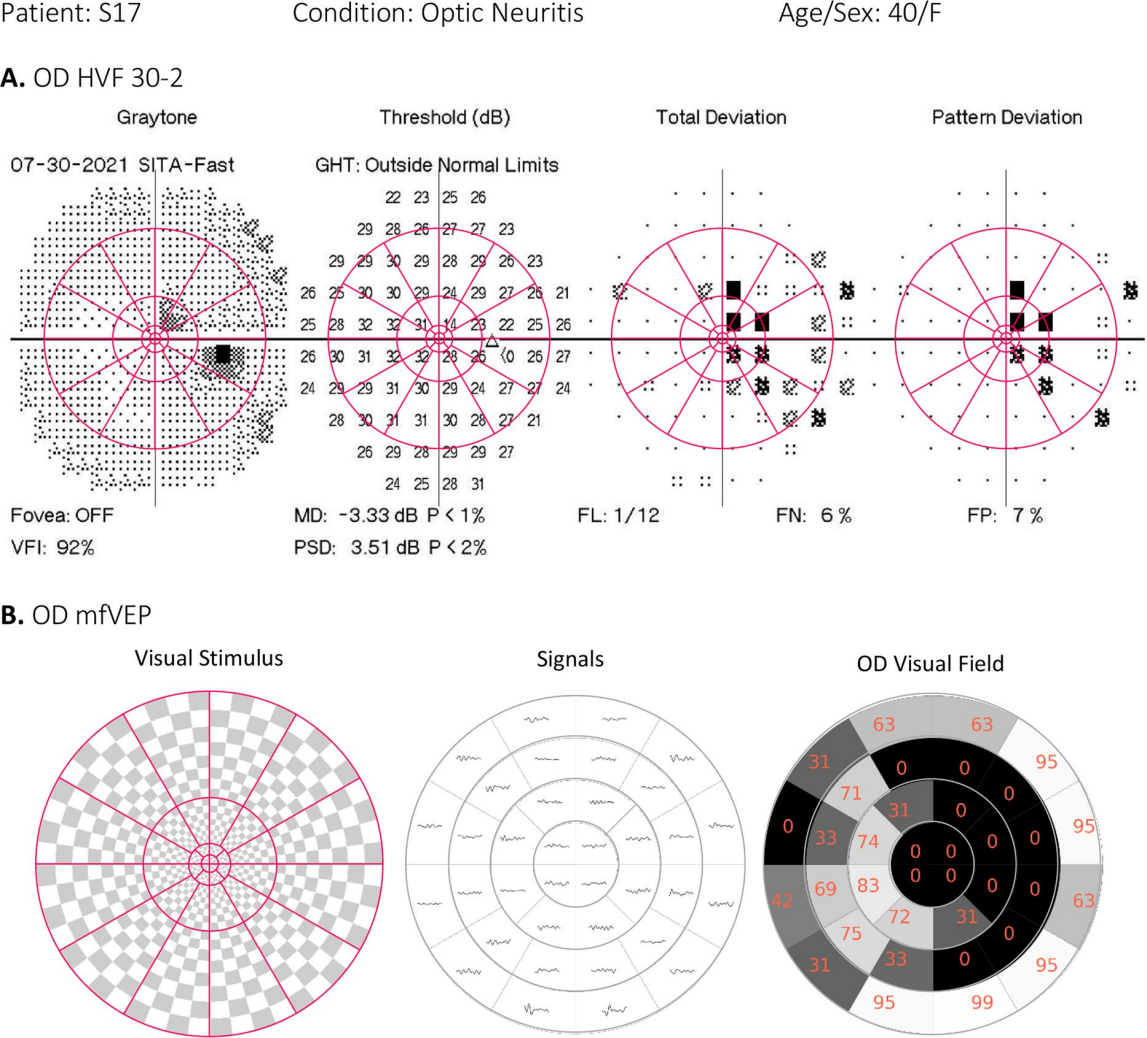
**A** HVF Central 30-2 Threshold Test with mfVEP sectors’ locations overlayed on top, **B** NeuroVEP mfVEP test for right eye (OD) of an ON subject. Brain MRI Results: Optic Neuritis, Optometrist Notes: Vision loss in right eye (OD), can see some colors, not shapes. HVF: Cecocentral Scotoma. NeuroVEP ffVEP: Abnormal waveform for the right eye (OD). Probably a prechiasmatic dysfunction. NeuroVEP mfVEP: Very poor or loss of vision in the black area

Figure 13 shows the comparison of the NeuroVEP’s mfVEP test results and the HVF (Humphrey Visual Field) test of subject S17. The HVF test is conducted with 30-2 standard, but we have overlayed the location of the 36-sector mfVEP stimulus (which better matches with HVF 24-2 standard test area) on the HVF results to be able to compare the two tests. Optic neuritis in the right eye (OD) of this subject was confirmed by MRI. The optometrist reports mention vision loss in OD and HVF confirms a cecocentral scotoma. The subject can distinguish colors in the right eye but cannot see shapes. This subject expressed extreme frustration with the HVF test but was very satisfied with the ease of compliance required by the NeuroVEP’s visual field test.

## Discussion

Diseases of the afferent visual system are central to the practice of neuro-ophthalmology (N-O). Right now, clinicians must perform screening using automated perimetry (AP) and optical coherence tomography (OCT) typically every 3–6 months for patients with such neuro-ophthalmic diseases. AP is subjective, susceptible to various artifacts, has poor accuracy and reliability, and does not provide crucial discriminatory information on the function of the neuro-optical pathways through the brain. OCT provides only structural information about the retina and optic disc (the proximal start of the optic nerve fibers). Visual function often dictates the type of treatment, but screening exams are a burden on already stretched neuro-ophthalmology clinics. Therefore, there is an unmet need for a practical, rapid, sensitive test that is objective and provides quantitative functional endpoints for early diagnosis and for monitoring patient response to newly developed treatments for neuro-ophthalmic diseases.

We have developed a new neuro-ophthalmic testing system called NeuroVEP that combines high-resolution sensors for scalp electric potentials and fields with precise visual stimuli in a portable, mobile computing enabled form-factor. NeuroVEP noninvasively measures VEP over the subject’s visual cortex, sensing its response to custom visual stimulus patterns presented on a head-mounted smartphone display. The neuroelectric recordings have been analyzed for full field stimulus (ffVEP) and 36 visual field sectors (mfVEP). The ff/mfVEP test is monocular, typically taking about 15 min to examine both eyes, and is completely objective, requiring no behavioral response from the subject—just fixation. Our study of ON/MS patients demonstrated the superiority of the NeuroVEP system over a conventional wired clinical VEP system in terms of portability and ease of use, superior VEP results, in addition to providing mfVEP visual field results which are classified by sector using machine learning. The mfVEP classifier results were evaluated using artificial defects introduced to the visual stimulus, achieving 91% accuracy on data unseen during training.

We validated the NeuroVEP’s system in a study of 20 subjects (10 healthy, 10 MS and/or ON patients). NeuroVEP’s ffVEP results were compared with the performance of the SOC VEP testing device at our partners’ clinic and the diagnosis reported by both systems agreed in 91% of cases. There are a couple of areas where the NeuroVEP device shines in comparison with the SOC device. The first benefit is convenience, both for the patient and the technician who sets up the device. The NeuroVEP device is a wireless headset that only needs a laptop computer to store the data. The technician just sets up the headset, starts the test, and the rest is done automatically. For the patient, the NeuroVEP device is comfortable and non-invasive. Our EEG electrodes are made of soft hydrogels that are held on the scalp with mild pressure, in contrast to the SOC, which uses needle electrodes inserted into the scalp. Also, comparing Fig. 10A and B, we can see other advantages of the NeuroVEP device starting with reliability: our results are based on many more trials which have been filtered with extensive denoising and signal processing methods to get a clear and reliable response. In contrast, the technician runs the SOC test several times with fewer trials and with varying results for the peak times and waveforms. The other key advantage of the NeuroVEP device is the fully automated analysis of the results immediately after the test. All parameters of the signals are validated against our normative dataset and color-coded based on the status of the responses. The NeuroVEP device also provides additional responses from 2 more locations on the scalp (O1 and O2), which can reveal additional insight into the defects on the visual pathway. Also, using multiple electrodes for each site increases the signal quality and reliability of the results.

Interpretation of anomalous mfVEP results is a challenging problem, previously left up to human experts, because signals from poorly sensed (or artificially blocked) sectors often contain residual neural activity, especially intrinsic alpha waves (8–13 Hz) and possibly other artifacts that can false-trigger simplistic signal quality metrics. Interocular comparison of mfVEPs has also been proposed [40]. The information about overlapping parts of the visual field from each eye is processed by collocal occipital regions. This often results in highly correlated spatial/temporal properties of monocular signals from healthy visual pathways; thus, significant differences in responses between eyes can be valid criteria for identifying damage to the pathways [41]. However, an obvious shortcoming of the interocular method is in cases where deficits exist in the pathways of both eyes. Incorporating several metrics derived from various unique features of responses while letting an automated ML model classify and score the responses can circumvent some of the shortcomings of simplistic signal metrics and especially improve the monocular analysis of the mfVEP results.

Where available, the NeuroVEP mfVEP results were in good agreement with Humphrey Automated Perimetry visual field analysis and with MRI and OCT findings. This pilot study indicates that NeuroVEP has the potential to be a reliable, portable, and objective diagnostic device for electrophysiology and visual field analysis for neuro-visual disorders.

Despite high accuracies achieved in both ffVEP and mfVEP tests, certain modifications can further improve the reliability, performance, and accuracy of the NeuroVEP device. For ffVEP and especially mfVEP tests, fixation is very important. Like any other visual field test, loss of fixation is detrimental to the test’s result. Therefore, an active eye tracker which tracks the gaze location during stimulation trials is a crucial add-on to the headset. Currently, we use only the live video feed from the Pupil Labs eye-tracking cameras inside the headset, but the test administrator is responsible for making sure the subject is compliant with the test’s protocols. Unfortunately, we found that this 3rd party eye-tracking device was cumbersome to set up and the data was often too noisy to be used for our purposes. A well-integrated eye-tracking solution that tracks the pupil direction, not only can eliminate the need for constant attention of the test administrator but also can provide a reliability metric for the test. Developing a reliability metric for the test, either based on the noise level of signals, alpha wave contamination, pupil location, or a fusion of all these parameters, is an important step in improving the NeuroVEP headset as a diagnostic apparatus. Increasing the number of stimulating sectors to 60 or more can increase the test’s resolution. Another promising area of improvement is the machine learning classification training procedure. In this study, we lumped together signals from various eccentricities in our machine learning training set. However, we do not get the same quality of signals from all eccentricities. Near peripheral ring sectors tend to produce higher SNRs, and the central sectors tend to produce lower ones; this discrepancy was also observed by other researchers [6]. Classifying all sectors using the same criteria can create a bias towards better responses in the periphery. Since we eventually use the classification probabilities for scoring the sector responses, a threshold moving algorithm can improve the slightly imbalanced classification problem. However, we believe obtaining more samples by testing more subjects (relatively small sample size is another limitation of this study) will enable us to break up the classification task into several sub-tasks using each set of sectors within the same eccentricity ring separately; this strategy may reduce biases and improve the overall accuracy. Furthermore, although interocular comparisons of the responses may have shortcomings if used as the singular criterion for the classification, being able to incorporate them in the ML framework should noticeably increase the accuracy of the classifications.

## Declarations

### Statement on the welfare of animals

no animals were used in this research
